# Combined depth imaging of choroid in uveitis

**DOI:** 10.1186/s12348-014-0018-8

**Published:** 2014-07-29

**Authors:** Padmamalini Mahendradas, Sumukh Madhu, Ankush Kawali, Indu Govindaraj, Poornachandra B Gowda, Anand Vinekar, Naren Shetty, Rohit Shetty, Bhujang K Shetty

**Affiliations:** Department of Uveitis and Ocular Immunology, Narayana Nethralaya Super Speciality Eye Hospital and Post Graduate Institute of Ophthalmology, 121/C, Chord Road, Rajaji Nagar 1st ‘R’ Block, Bangalore, 560010 India

**Keywords:** Uveitis, Optical coherence tomography, OCT, Spectral-domain optical coherence tomography (SD-OCT), Enhanced depth imaging, EDI, Combined depth imaging, CDI

## Abstract

**Background:**

Understanding the changes that occur in the choroid is of paramount importance in various uveitis entities. B-scan ultrasonography and indocyanine green angiography can be used to study choroid. Currently, spectral-domain optical coherence tomography is used as the standard noninvasive technique to study the choroid by enhanced depth imaging.

Our aim was to study the structural visibility of the choroid using spectral-domain optical coherence tomography in the same area of interest in patients with uveitis with posterior segment manifestations using conventional, enhanced depth imaging (EDI), and combined depth imaging (CDI) techniques.

**Results:**

Fifty-eight (58) eyes of 48 patients between age group 9 and 82 years were confirmed cases of uveitis. Out of the 48 patients, 21 (43.75%) were males while 27 (56.25%) were females. Sixteen eyes (27.59%) had intermediate uveitis, 33 (56.9%) had posterior uveitis, and 9 eyes (15.51%) had panuveitis.

For posterior vitreous, there was substantial agreement for all the three groups (kappa value of 0.77, 0.73, and 0.72 in groups 1, 2, and 3, respectively). For vitreo retinal interface and inner choroid, there was perfect interobserver agreement, and for outer choroid, there was substantial to almost perfect interobserver agreement (kappa value of 0.71, 0.81, and 0.86 in groups 1, 2, and 3, respectively).

Chi-squared test was done to compare the three groups. The method of scanning had a significant effect on the visualization of posterior vitreous and the outer choroid (*p* < 0.01) and did not have an effect on the visualization of vitreoretinal interface, inner retina, outer retina, and inner choroidal layers (*p* > 0.05).

**Conclusion:**

The CDI technique alone might provide a good structural visibility compared to normal and EDI scanning done separately in patients with uveitis with posterior segment pathology. CDI OCT technique is thus able to visualize all posterior structures in a single image in patients with uveitis with posterior segment manifestations.

**Electronic supplementary material:**

The online version of this article (doi:10.1186/s12348-014-0018-8) contains supplementary material, which is available to authorized users.

## Background

The choroid is a highly vascularized and pigmented tissue which extends from the ora serrata anteriorly to the optic nerve posteriorly [[1]]. An understanding of choroidal pathology is critical for an accurate assessment of many posterior segment changes in uveitis. Evaluation of the choroid can be done by indocyanine green (ICG) angiography [[2],[3]], laser Doppler flowmetry [[4]], ultrasound, and optical coherence tomography (OCT) [[5]-[7]].

ICG is considered the gold standard for the evaluation of choroidal pathology. However, it has a major disadvantage of being an invasive technique accompanied by harmful effects related to the indocyanine green dye experienced by few [[2],[3]]. OCT has been the gold standard noninvasive technique to visualize fine retinal structural changes for many ocular diseases. Earlier, adequate morphologic examination of the choroid using OCT was not possible mainly due to the presence of pigments in the RPE layer which attenuate the incident light and also due to its posterior location. Recent reports however demonstrated successful examination and measurement of choroidal thickness in normal and pathologic states using spectral-domain optical coherence tomography (SD-OCT) instruments [[5]-[10]].

In the SD-OCT, both reflected beams of light are compared and combined into an interference pattern by the spectral interferogram or spectrometer which is a modified Michelson interferometer [[11],[12]]. Fourier equations transform this spectral interferogram into two OCT mirror images. The screen of the OCT instrument depicts one of these two images. The vitreous is seen at the top of the screen, while the choroid is seen at the bottom of the screen.

In the conventional scan, the vitreous is at the peak of the OCT sensitivity curve and the closest to the point of maximum sensitivity, called as the zero delay line [[13],[14]], whereas the choroid is far from the zero line. Hence, there is a good visibility of posterior vitreous, whereas with increasing depth into the tissue, the signal is reduced and choroidal visibility is poor.

More recently, the ability to visualize the choroidal anatomic features has been improved with the development of the enhanced depth imaging (EDI) technique on SD-OCT [[15]-[17]]. In this technique, the OCT instrument is positioned closer to the eye due to which an inverted mirror image is obtained and choroid now becomes closer to the zero delay line than the vitreous. The choroidal visibility enhances compared to the noninverted image. However, the posterior vitreous visibility is affected.

To overcome this imaging limitation and to obtain a single comprehensive image of both the vitreoretinal interface and choroid, Barteselli et al. in 2013 [[18],[19]] developed a novel imaging method called the combined depth imaging technique using a commercially available SD-OCT device. The study tested the ability of the technique to visualize vitreoretinal and choroidal structures in a series of normal eyes and eyes with cataract.

Here in our study, we have used the combined depth imaging (CDI) technique to visualize posterior segment structures in uveitis patients with posterior segment manifestations. The main aim of the study was to assess the structural visibility of the posterior vitreous, vitreoretinal interface, and the inner and outer choroidal borders in patients having uveitis with posterior segment manifestations using the CDI technique. The results obtained were compared with conventional and EDI techniques. Our objective was to assess whether a single comprehensive image obtained by this technique is comparable the conventional and EDI images taken separately.

## Methods

Institutional ethics committee approval was obtained to conduct the cross-sectional observational case series of SD-OCT findings in posterior segment changes in uveitis patients. The study was conducted at the Department of Uveitis and Ocular Immunology, Narayana Nethralaya Super Speciality Eye Hospital and Post Graduate Institute of Ophthalmology, Bangalore, in adherence to the tenets of the Declaration of Helsinki.

Fifty-eight eyes of 48 patients diagnosed as uveitis with posterior segment manifestations were included in the study. In patients having bilateral disease, both eyes were included. Exclusion criteria included known uveitis patients with significant vitritis or any anterior segment or media opacity due to which it would be difficult to obtain a clear OCT scan. After obtaining written informed consent to participate in this research, all subjects underwent initial slit lamp examination followed by dilatation with tropicamide eye drops. Patients clinically diagnosed as having uveitis with posterior segment manifestations were then subjected to color fundus photography and OCT scans (high-definition SD-OCT using Spectralis™ (Heidelberg Engineering GmbH, Heidelberg, Germany) using conventional, EDI, and CDI techniques in all cases with fluorescein angiography and indocyanine green angiography in selected cases.

### Combined depth imaging technique

Barteselli et al. in 2013 [[19]] had described the CDI technique in detail. Here, we provide you a brief overview of the same. The CDI technique is an image process modification that combines conventional SD-OCT scans with EDI OCT scans into a single image. While using this technique, the vitreoretinal interface is enhanced in the first half of the scanning process followed by enhancement of choroid in the other half. Thus, over an average of 100 separate OCT scans, the vitreoretinal interface is highly enhanced in the first 50 scans. The operator then selects the EDI button; subsequently in the next 50 scans, the choroid becomes highly enhanced. The device later merges conventional scans with EDI OCT scans into a single comprehensive image with good sensitivity throughout the imaging process [[19]].

### Optical coherence tomography scanning protocol

The imaging for all the patients included in the study was done by a single experienced technician. The Spectralis HRA was set to perform a 9-mm high-resolution horizontal B-scan, centered on the area of interest. An internal fixation light was used to center the scanning line on the area of interest. A horizontal or a vertical linear scan was obtained depending upon the area of interest along with the raster scan for each patient. The averaging system was set to 100 OCT scans. A sequence of three different images was performed for each eye of the patients, making sure that the same area was imaged in all three techniques.

After ensuring proper positioning and comfort of the patient, the operator began the scanning by positioning the OCT scan at the upper half of the screen. The operator then activated the averaging system of the device, and after reaching at least 50% of the averaging, the image was captured. The image obtained by this process was the conventional OCT image.

The position of the OCT scan was now shifted to the lower half of the screen. The operator pressed the EDI button to activate the EDI acquisition software. After reaching at least 50% of the averaging, the image was captured. The image obtained by this technique was the EDI OCT image.

Now, the position of the scan was shifted to the middle of the screen. The operator activated the averaging system of the device and the image was captured. After reaching 50% of the averaging, the operator pressed the EDI button and activated the EDI acquisition software. As soon as a good quality image was seen, the image was captured. The image obtained by this technique was the CDI OCT image.

### Optical coherence tomography imaging analysis

The three images for each eye at the same area of interest were taken. Patient information and the type of scanning technique as well as the images were masked and mixed randomly. Two independent masked physicians reviewed each image on the same monitor with same resolution at different time points and graded the visualization of posterior vitreous cavity, vitreoretinal interface*,* the inner border of the choroid, and the outer border of the choroid separately. Grade 0 indicated that the analyzed area was not visible; grade 1 indicated that the border was barely visible, and grade 2 indicated that the border was clearly visible.

The interobserver agreement for the grading of the posterior vitreous, vitreoretinal interface, the inner border, and the outer border of the choroid was assessed using the Cohen κ. Chi-squares test was used to compare the grading of the posterior vitreous, vitreoretinal interface, inner border, and the outer border of the choroid among the three OCT images for each eye. Statistical analysis was performed using the SPSS software (SPSS 17.0).

### Consent

Written informed consent was obtained from the patients for publication.

## Results

Fifty-eight (58) eyes of 48 patients between age group 9 and 82 years (median 45 years) were confirmed cases of uveitis. Twenty-one (43.75%) out of the 48 patients were males while 27 (56.25%) were females. Sixteen eyes (27.59%) had intermediate uveitis, 33 (56.9%) had posterior uveitis, and 9 eyes (15.51%) had panuveitis (Table 1).

**Figure 1:**
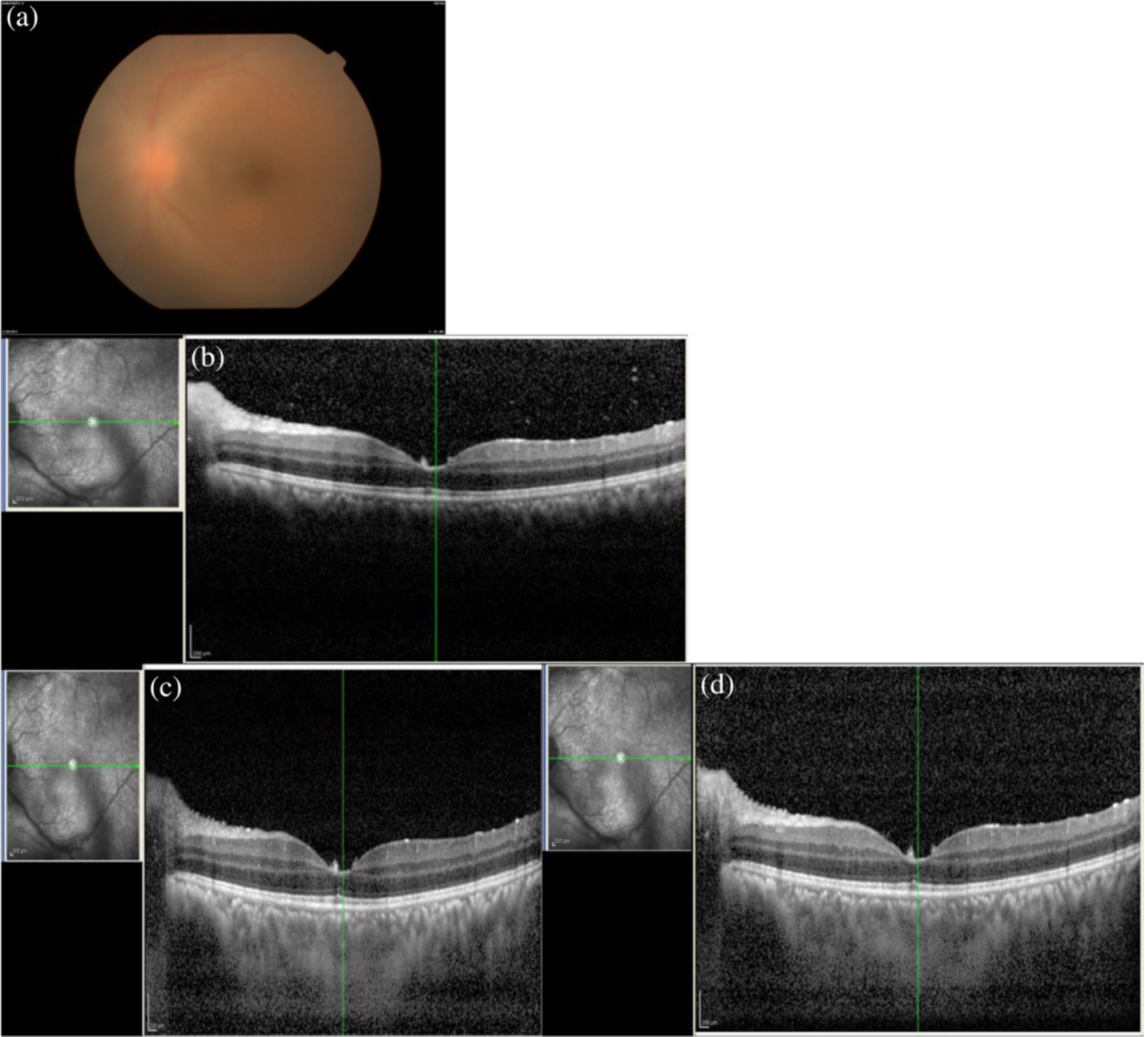
**Acute retinal necrosis with posterior uveitis. (a)** The fundus photo of a patient with acute retinal necrosis with vitritis. This example demonstrates the limitation of the EDI **(c)** and CDI **(d)** techniques in obtaining a good posterior vitreous visibility in patients with dense vitritis where the normal OCT scan can pick up posterior vitreous cells as can be seen in **(b)**.

**Figure 2:**
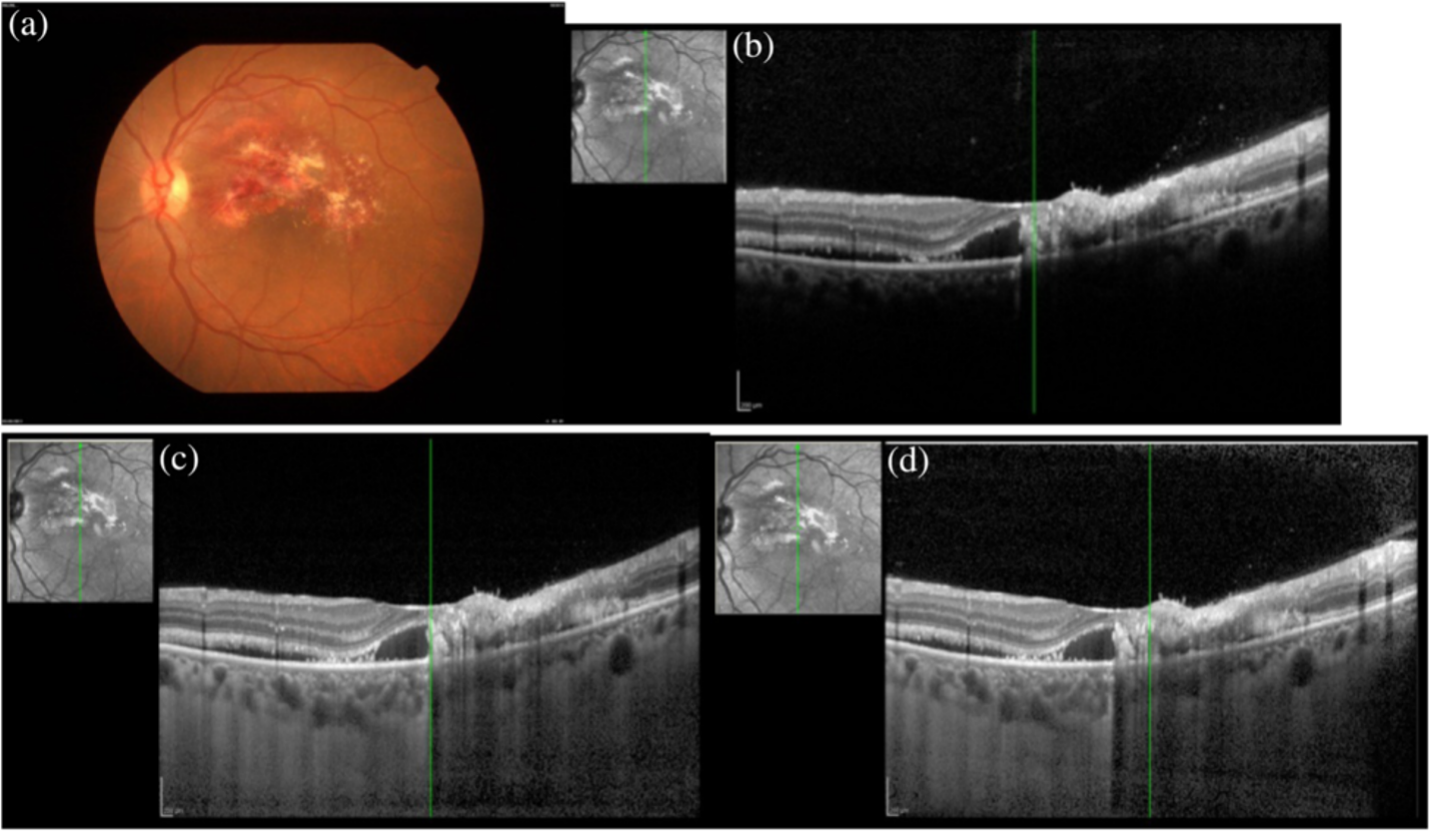
**CMV retinitis. (a)** The fundus seen shows the left eye of a female diagnosed with CMV retinitis. The posterior pole shows well demarcated area of hemorrhages with retinitis. The **(b)** conventional, **(c)** EDI, and **(d)** CDI techniques.

**Figure 3:**
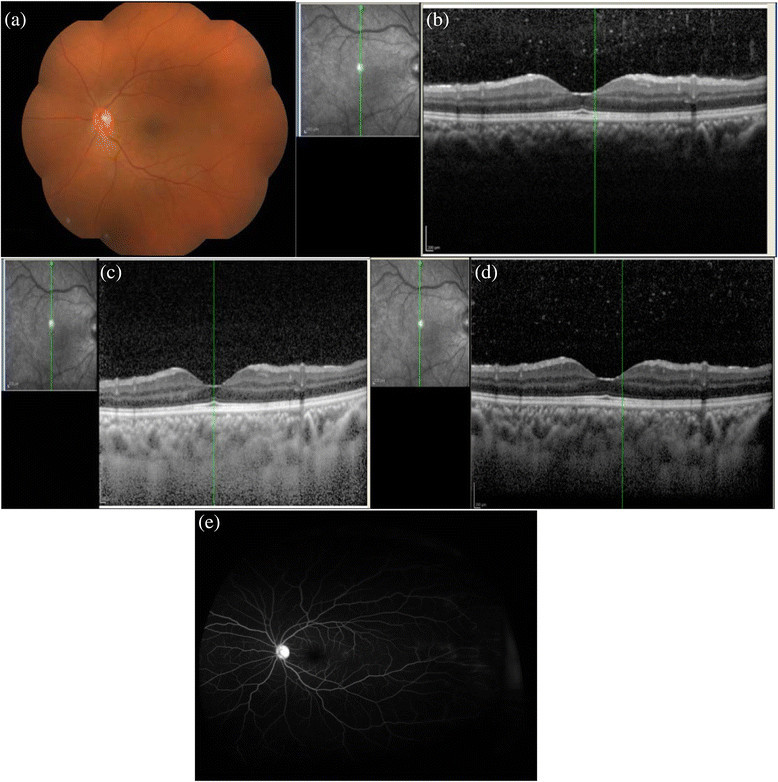
**Retinal vasculitis. (a)** The fundus photo of the left eye of a patient with retinal vasculitis which looks essentially normal with hazy media. However, her fluorescein angiography demonstrated areas of perivascular leak in midperiphery and periphery **(e)**. **(b)** Conventional OCT scan shows presence of abundant vitreous cells. The outer border of the choroid is not visible. **(c)** EDI scan allows good visibility of both the inner and outer choroidal borders, but the posterior vitreous shows no cells. However, the **(d)** CDI scan shows both the presence of vitreous cells with good choroidal visibility.

Different etiologies such as tuberculosis (12 cases), idiopathic uveitis (8), Vogt-Koyanagi-Harada (VKH) disease (4), acute retinal necrosis (Figure 1) and viral retinitis with herpes simplex infections (4), sarcoidosis (3), toxoplasmosis (2), cytomegalovirus retinitis (Figure 2) (2), Behcet's disease (2), bacterial endophthalmitis (2), idiopathic retinal vasculitis (Figure 3) (2) with vascular occlusion (2), systemic lupus erythematosis (1), multiple sclerosis (1), sympathetic ophthalmia (1), uveitis with RP (1), and rheumatoid arthritis (1) were studied.

**Table 1:** **Demographic profile of the uveitis cases**

Total no of cases		48
Total no of eyes		58
Laterality	Unilateral	38
	Bilateral	10
Sex	Male	21 (25 eyes)
	Female	27 (33 eyes)
Age group		9-82 (Median -45 yrs)
	Intermediate uveitis	16 eyes (27.59%)
Anatomical diagnosis	Posterior uveitis	33 eyes (56.9%)
	Panuveitis	9 eyes (15.51%)

A kappa value of more than 0.7 is considered substantial for a given study. For posterior vitreous, there was substantial agreement for all the three groups (kappa value of 0.77, 0.73, and 0.72 in groups 1, 2, and 3, respectively) (Table 2). For vitreo retinal interface and inner choroid, there was perfect interobserver agreement; for outer choroid, there was substantial to almost perfect interobserver agreement (kappa value of 0.71, 0.81, and 0.86 in groups 1, 2, and 3, respectively) [[20]].

**Table 2:** **Interobserver agreements between conventional, EDI and CDI scans**

Area	Weight kappa for conventional OCT	Weight kappa for EDI OCT	Weight kappa for CDI OCT
Posterior vitreous	0.771	0.732	0.722
Vitreoretinal interface	1	1	1
Inner choroid	1	1	1
Outer choroid	0.718	0.809	0.865

Chi-squared test was done to compare the three groups. The method of scanning had a significant effect on the visualization of the posterior vitreous and the outer choroid (*p* < 0.01) and did not have an effect on the visualization of vitreoretinal interface, inner retina, outer retina, and inner choroidal layers (*p* > 0.05). With conventional technique, the outer choroid was not visualized, (Additional file 1: Figure S1), and with enhanced depth imaging, the posterior vitreous surface was not clearly visualised (Additional file 2: Figure S2). However, with combined depth imaging, it is possible to visualize the posterior vitreous and the outer choroid simultaneously.

## Discussion

**Figure 4:**
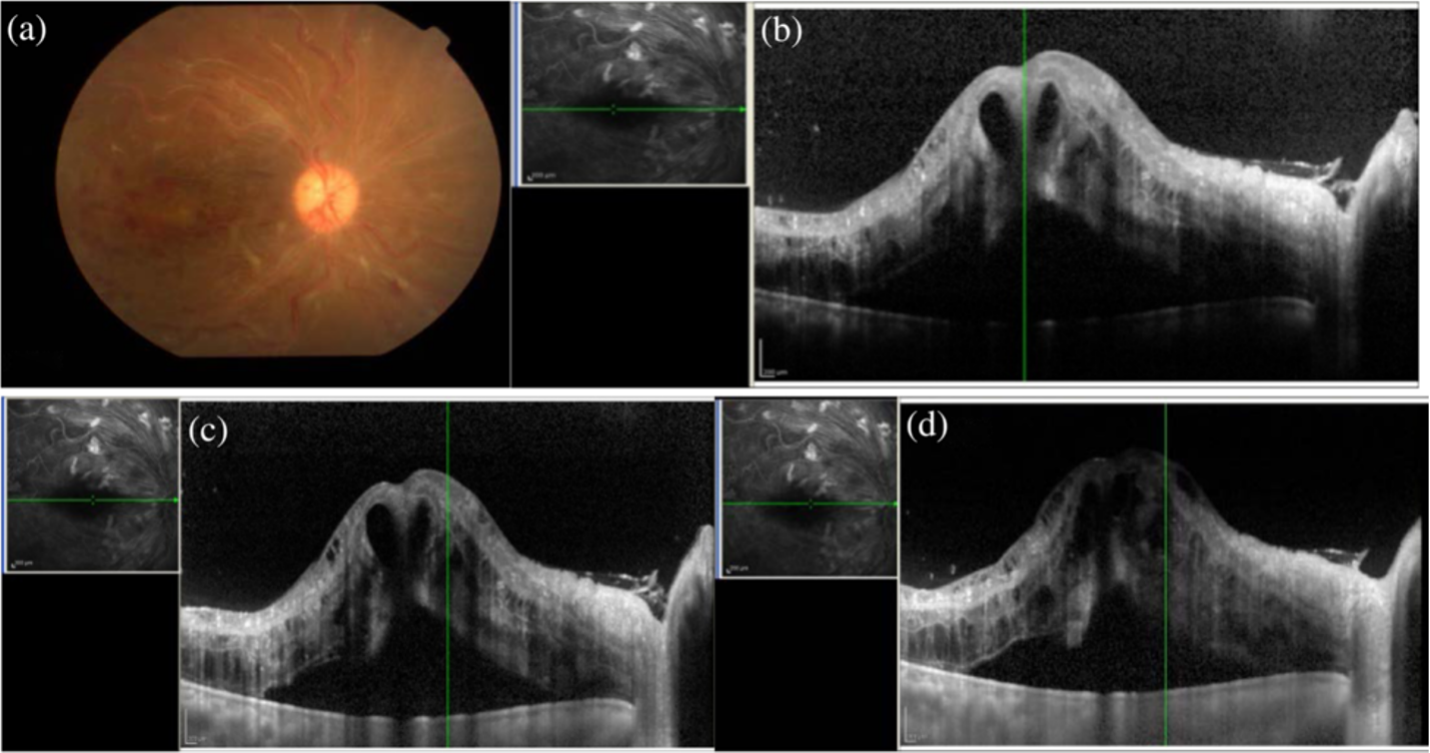
**Systemic lupus erythematosus with cystoid macular edema.** A 20-year-old female diagnosed with systemic lupus erythematosus was seen by us for ophthalmic evaluation. **(a)** The fundus picture of the right eye shows gross cystoid macular edema with multiple retinal hemorrhages and increased tortuosity of the retinal vessels. The **(b)** conventional, **(c)** EDI, and **(d)** the CDI scans taken over the same horizontal area of interest. This example illustrates the limitation of the CDI technique in not visualizing the outer choroidal border in cases of increased retinal thickness.

This study demonstrated the structures seen in the conventional, EDI, and CDI techniques in the posterior segment pathologies of various uveitis entities. In this study, the CDI OCT technique was able to visualize all posterior structures in a single image in patients with uveitis with posterior segment manifestations. The new imaging technique is simple and fast to perform except in patients with significant media opacity where scan quality is hampered significantly and in patients with increased retinal thickness as explained in a case example of central retinal vein occlusion with macular edema in a case of systemic lupus erythematosis (Figure 4).

The single scan of a particular area, obtained with conventional SD-OCT and EDI OCT separately, may not precisely image the exactly same area, because after collecting the first image, the patient may change the fixation slightly [[19]]. Because of this small movement of the fixating point, the recorded image may not correspond to the same area. The CDI technique is useful in having a complete evaluation of the structures of the same area of interest, without being affected by fixation changes.

**Figure 5:**
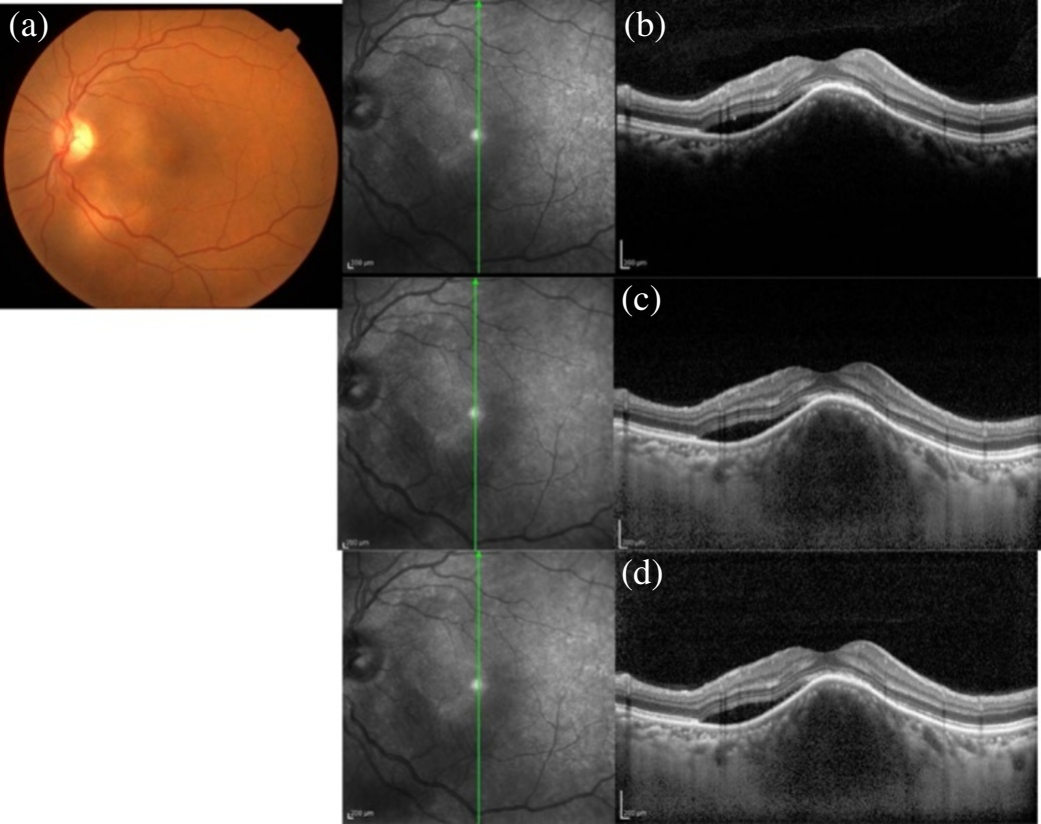
**Tuberculosis.** A 46-year-old female has got presumed ocular tuberculosis. **(a)** The color fundus photograph of the left eye shows inferotemporal choroidal granuloma with localized serous retinal detachment. **(b)** Conventional OCT scan shows the presence of posterior vitreous detachment with hyporeflective area in the choroid with serous retinal detachment (RD). The posterior extent of the hyporeflective area could not be made out. **(c)** The EDI technique clearly demarcates the posterior border of the hyporeflective with serous RD. However, the visibility of the posterior vitreous is compromised. **(d)** The CDI technique demonstrates the entire extent of the hyporeflective area with RD and without significantly compromising the posterior vitreous visibility.

Inflammatory CNVM was seen in two patients. Both the cases were associated with tuberculous uveitis. One was associated with active tuberculous uveitis, and other one was seen in healed TB uveitis. In tuberculosis uveitis, the cells in the posterior vitreous were visible which was made out well only in conventional and CDI scans. Serous retinal detachment, outer retinal changes in form of disruption of IS/OS junction, proliferating retinal pigment epithelium cells [[21]] were well made out in all three scanning techniques, whereas increased choroidal thickness with hyporeflective choroidal granulomas [[22]] in cases of choroiditis [[22]] and outer choroidal border was well made out only in the EDI and CDI scans (Figure 5).

**Figure 6:**
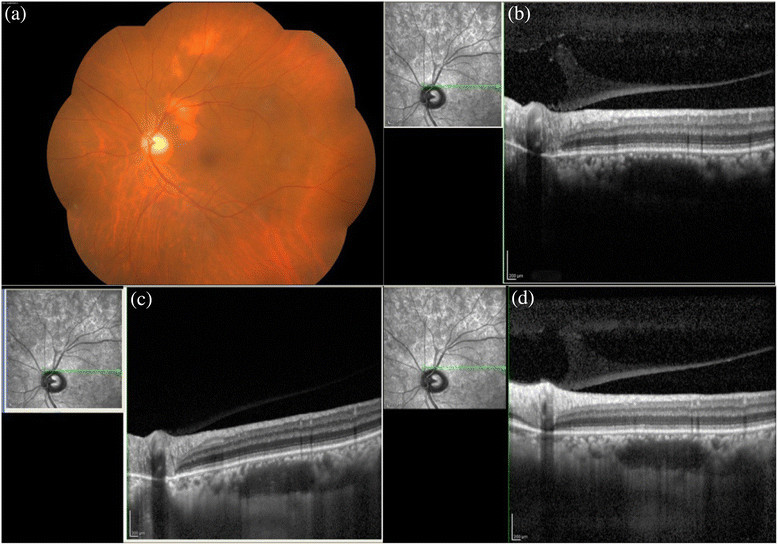
**Sarcoidosis.** A 63-year-old female with confirmed case of sarcoidosis. **(a)** The color fundus montage photograph of the left eye shows an orange-yellow lesion superotemporal to the optic disc, suggestive of a choroidal granuloma. **(b)** Conventional OCT scan shows the presence of posterior vitreous detachment with hyporeflective area in the choroid. The posterior extent of the hyporeflective area could not be made out. **(c)** The EDI technique clearly demarcates the posterior border of the hyporeflective area. However, the visibility of the posterior vitreous is compromised to a certain extent. **(d)** The CDI technique demonstrates the entire extent of the hyporeflective area without significantly compromising the posterior vitreous visibility.

In sarcoidosis, localized hyporeflective choroidal thickening in the areas of choroidal granulomas was noticed, and our observation was similar to that by Rostaqui et al. [[23]]. Also, the outer border of the choroid was seen clearly in the EDI and CDI scans (Figure 6). Nakai et al. [[24]] noticed an increased choroidal thickness in VKH disease which progress over time. Patients with long-standing VKH disease had thinner choroids as per the study of Da Silva et al. [[25]].

**Figure 7:**
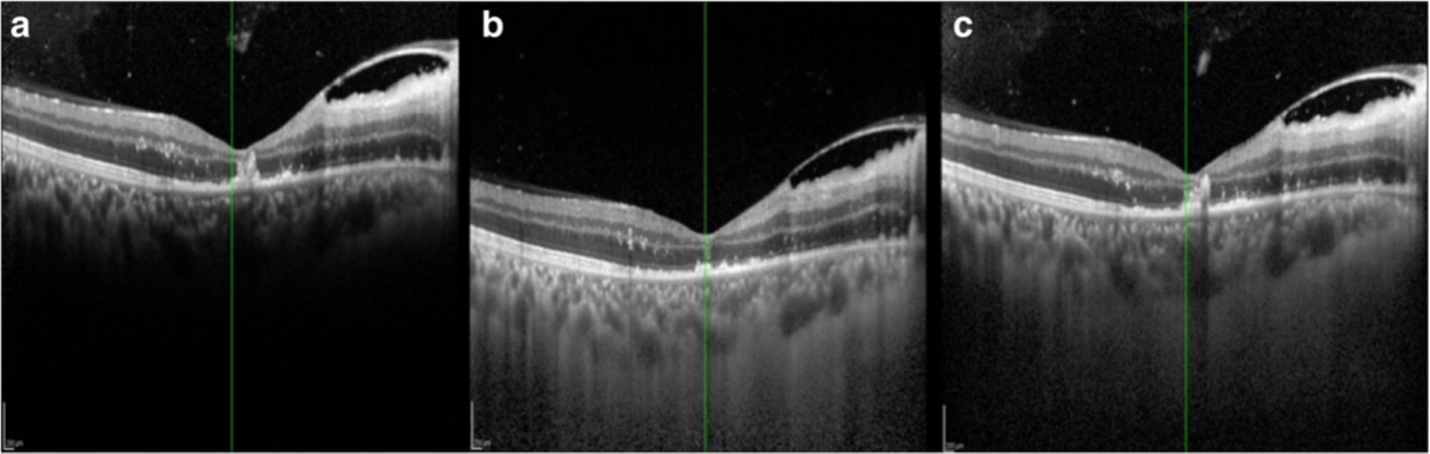
**Healing toxoplasmic retinitis.** The **(a)** conventional, **(b)** EDI, and **(c)** CDI scans in serial order. Single CDI scanning over a combination of both shows good simultaneous visibility of both the posterior vitreous and outer choroidal border.

**Figure 8:**
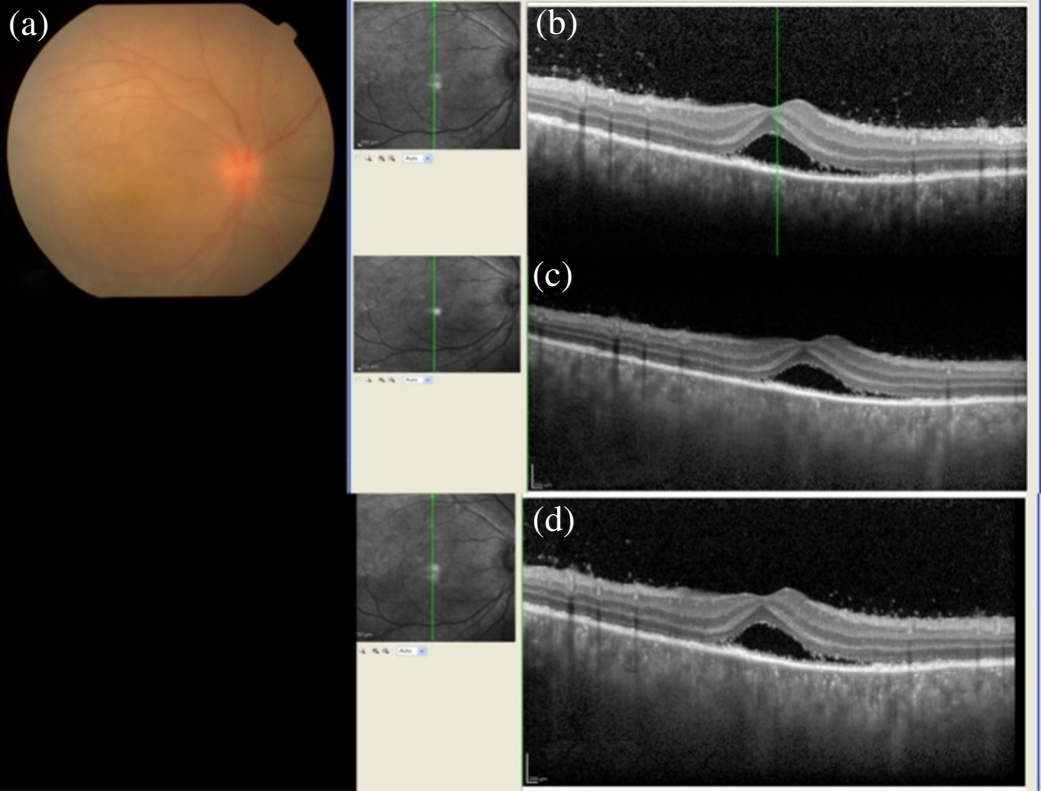
**Sympathetic ophthalmia.** **(a)** The color fundus photograph of the right eye shows hyperemic disc with localized serous retinal detachment. The **(b)** conventional, **(c)** EDI, and **(d)** CDI scans in serial order. **(b)** The conventional OCT scan shows the presence of posterior vitreous cells with retinal detachment, and the outer border of the choroid is not visible. (c) EDI shows poor visibility of the posterior vitreous with good visibility of the choroid, and the outer choroid border is well made out. **(d)** CDI shows the presence of posterior vitreous cells with serous retinal detachment with good visibility of the choroid with the outer choroid border.

In toxoplasmic retinochoroiditis, OCT shows thickening and disorganization of both the retina and underlying choroid in acute stages [[26]] followed by scar formation with thinning of the retina often in conjunction with irregularity of the outer retinal layers as seen in one of our cases (Figure 7). Gupta et al. [[27]] demonstrated the reversible changes of serous retinal detachment with photoreceptor layer involvement during the acute phase of sympathetic ophthalmia by SD-OCT. The use of the CDI technique to study the changes in the posterior vitreous and retinal and choroidal layers in acute cases of sympathetic ophthalmia has been demonstrated in our study (Figure 8).

**Figure 9:**
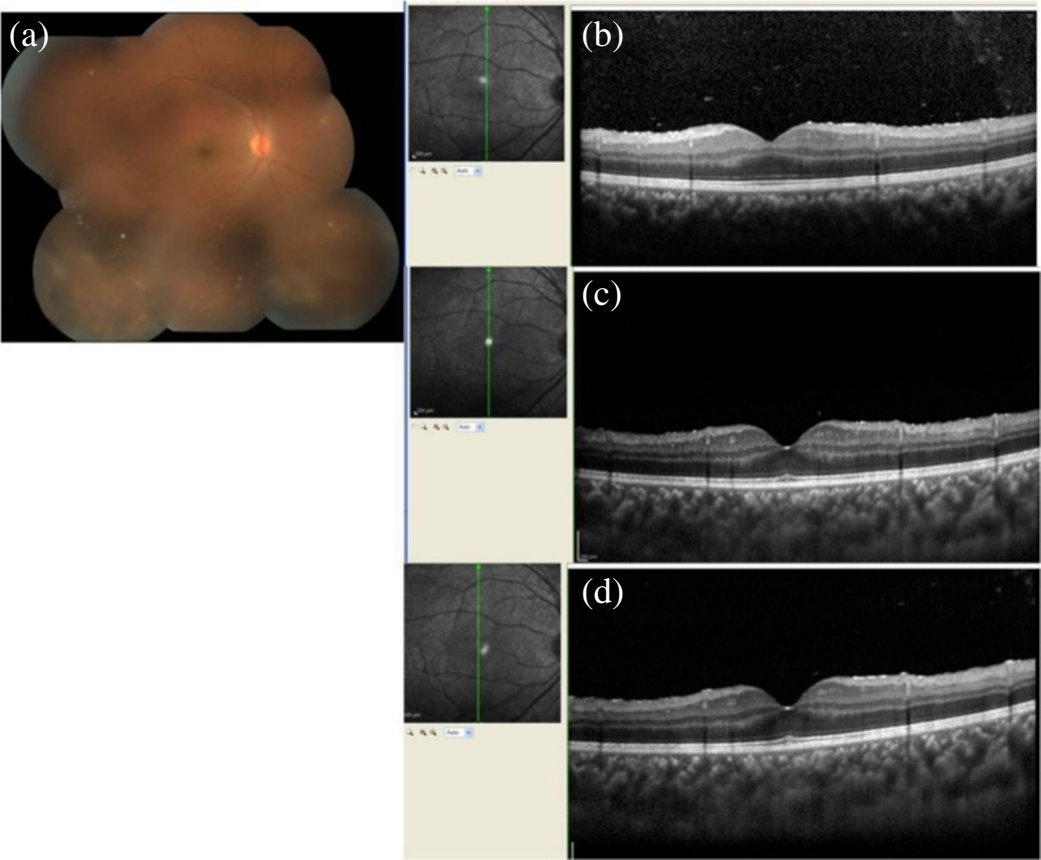
**Behcet's panuveitis.** **(a)** The fundus photo of the right eye of a patient with Behcet’s disease. The **(b)** conventional, **(c)** EDI, and **(d)** CDI scans. The conventional OCT scan shows the presence of posterior vitreous cells, and the outer border of choroid is not visible. EDI shows poor visibility of the posterior vitreous with good visibility of the choroid. CDI shows the presence of posterior vitreous cells with good visibility of the choroid with the outer choroid border.

We also noticed choroidal thickening during the active phase of Behcet's posterior uveitis (Figure 9), and our observation was similar to that in the study of Kim et al. [[28]]. The outer border of the choroid in Behcet's uveitis was well made out in the EDI and CDI scans.

Overall, the CDI technique does offer a single comprehensive image based on which both the posterior vitreous and outer choroid can be studied. However, our study as well as the CDI technique has its own limitations. Though the inner and outer choroidal border visibility in different uveitis entities has been studied, each layer of the choroid needs to be evaluated separately with choroidal thickness measurements. Furthermore, the CDI technique is not possible in patients with poor fixation. As mentioned in the example in Figure 4, the outer choroidal visibility is affected in patients with increased retinal thickness, and CDI scan is not helpful. Also, the technique is possible only with the Spectralis HRA and no other commercially available SD-OCT and is possible only with linear scan and not with the raster scan [[19]].

## Conclusion

In conclusion, the manual technique of CDI OCT is easy, fast, and sensitive enough to visualize posterior vitreoretinal and choroidal structures together in a single image in case of uveitis with posterior segment manifestations using a commercially available and widely used SD-OCT device. Dedicated built-in software may be useful to obtain this full-depth imaging result automatically. However, ICG still remains the gold standard imaging modality for choroidal pathology. Our study aims at assessing only the structural visibility using the CDI technique, and further studies are required to prove its efficacy in monitoring the progression of uveitis with posterior segment manifestations.

## Additional files

## Electronic supplementary material

Additional file 1: Figure S1.: Bar diagram showing the effect of method of scanning on visualization of the outer choroid. Enhanced depth imaging and combined depth imaging enable visualization of the outer choroid which is not possible in normal OCT scan. (DOC 45 KB)

Additional file 2: Figure S2.: Bar diagram showing the effect of method of scanning on visualization of the posterior vitreous surface. With enhanced depth imaging, the posterior vitreous surface is not visualized, whereas in the combined depth imaging, both the posterior vitreous surface and the outer choroid are visualized. (DOC 48 KB)

Below are the links to the authors’ original submitted files for images.

Authors’ original file for figure 1

Authors’ original file for figure 2

Authors’ original file for figure 3

Authors’ original file for figure 4

Authors’ original file for figure 5

Authors’ original file for figure 6

Authors’ original file for figure 7

Authors’ original file for figure 8

Authors’ original file for figure 9
