# Supplementary figures and images for: Combined depth imaging of choroid in uveitis
Source: J Ophthalmic Inflamm Infect. 2014 Jul 29;4:18. doi: 10.1186/s12348-014-0018-8 (PMC4883994; doi:10.1186/s12348-014-0018-8)

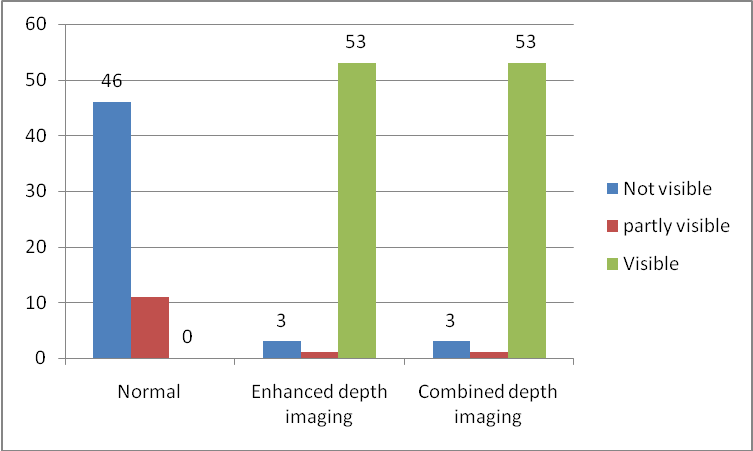

Supplement: Supplementary file 1 — Additional file 1: Figure S1.: Bar diagram showing the effect of method of scanning on visualization of the outer choroid. Enhanced depth imaging and combined depth imaging enable visualization of the outer choroid which is not possible in normal OCT scan. (DOC 45 KB) [file 12348_2014_18_MOESM1_ESM.doc]

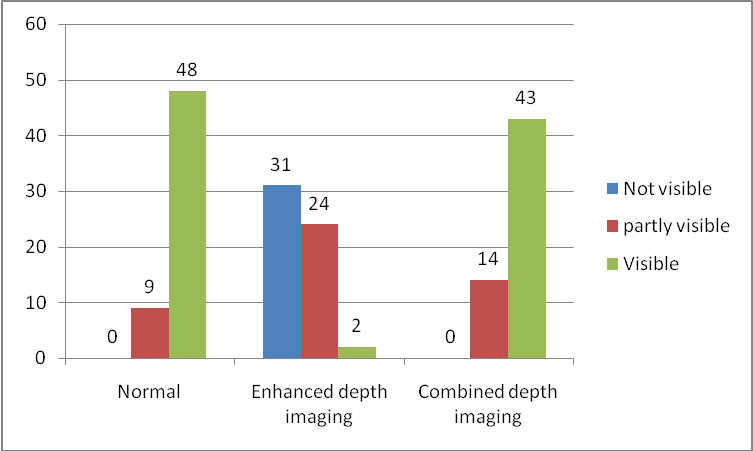

Supplement: Supplementary file 2 — Additional file 2: Figure S2.: Bar diagram showing the effect of method of scanning on visualization of the posterior vitreous surface. With enhanced depth imaging, the posterior vitreous surface is not visualized, whereas in the combined depth imaging, both the posterior vitreous surface and the outer choroid are visualized. (DOC 48 KB) [file 12348_2014_18_MOESM2_ESM.doc]

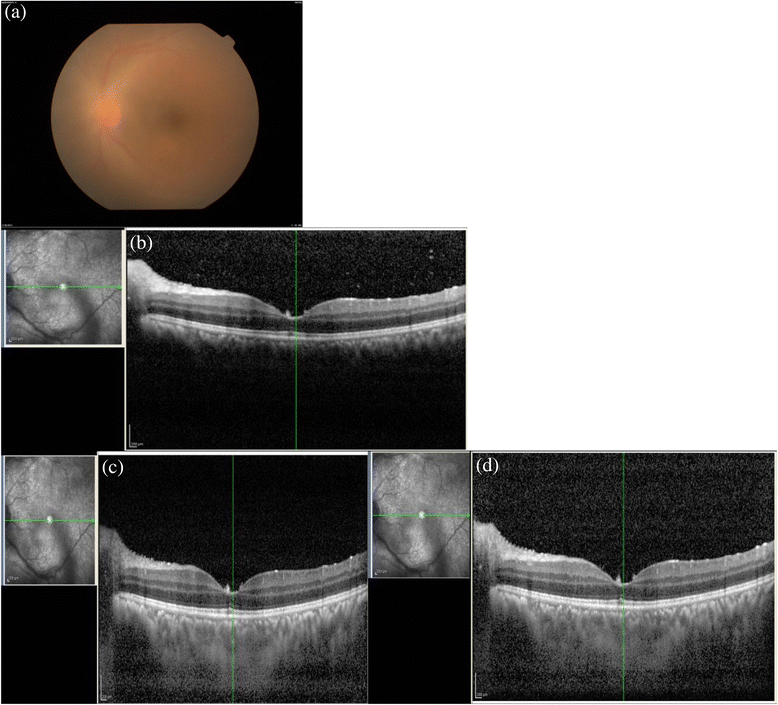

Supplement: Supplementary file 3 — Authors’ original file for figure 1 [file 12348_2014_18_MOESM3_ESM.gif]

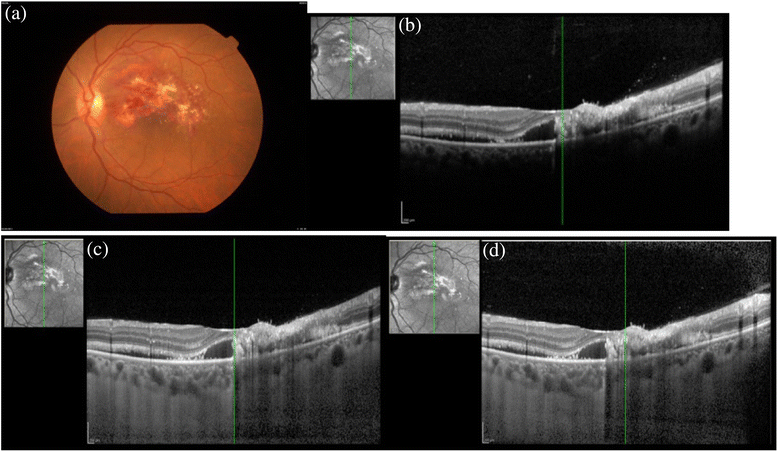

Supplement: Supplementary file 4 — Authors’ original file for figure 2 [file 12348_2014_18_MOESM4_ESM.gif]

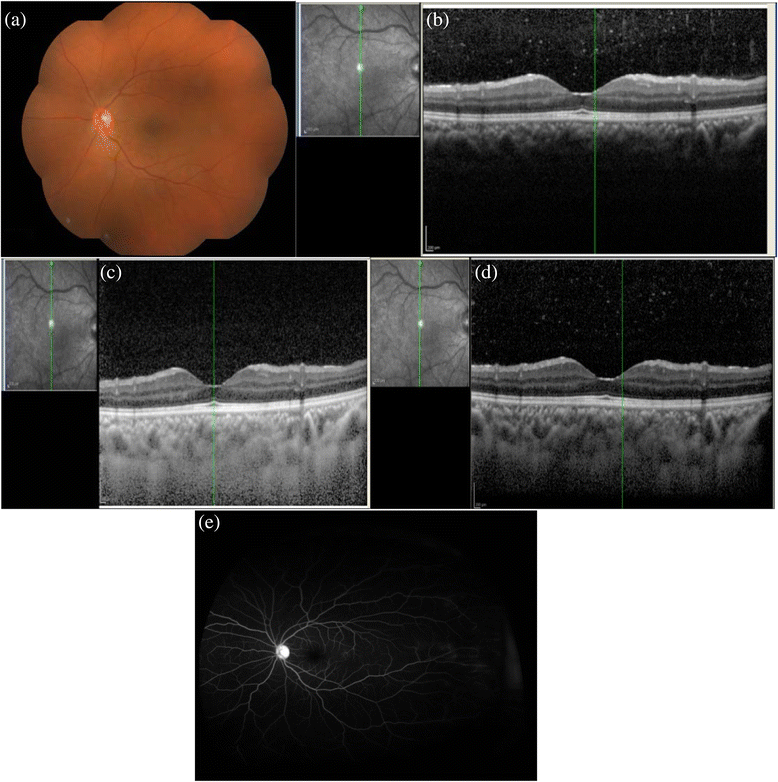

Supplement: Supplementary file 5 — Authors’ original file for figure 3 [file 12348_2014_18_MOESM5_ESM.gif]

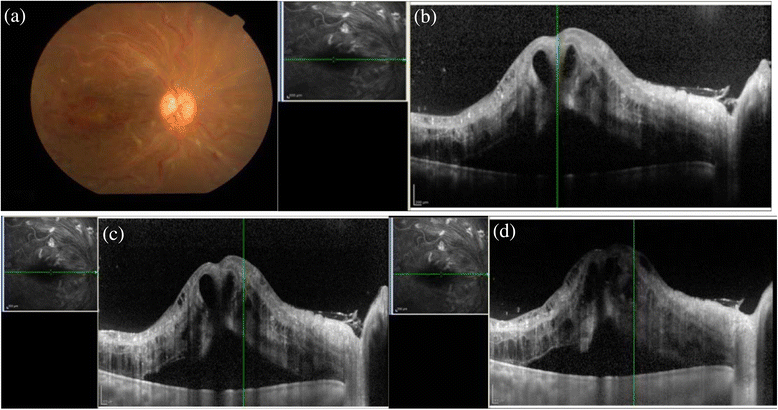

Supplement: Supplementary file 6 — Authors’ original file for figure 4 [file 12348_2014_18_MOESM6_ESM.gif]

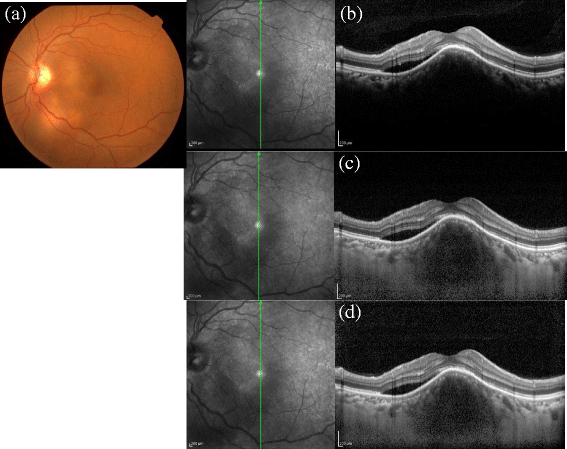

Supplement: Supplementary file 7 — Authors’ original file for figure 5 [file 12348_2014_18_MOESM7_ESM.gif]

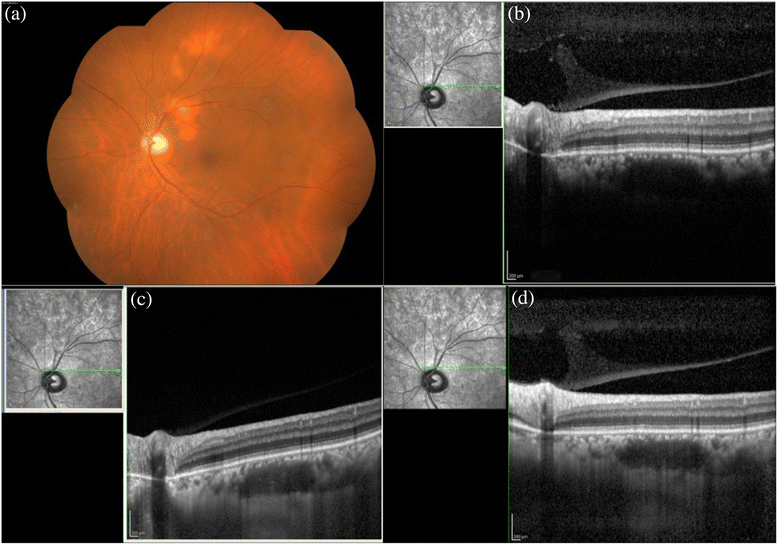

Supplement: Supplementary file 8 — Authors’ original file for figure 6 [file 12348_2014_18_MOESM8_ESM.gif]

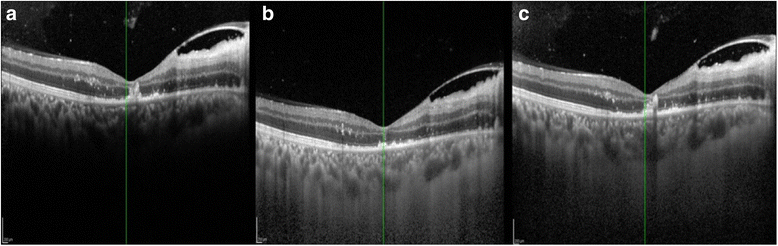

Supplement: Supplementary file 9 — Authors’ original file for figure 7 [file 12348_2014_18_MOESM9_ESM.gif]

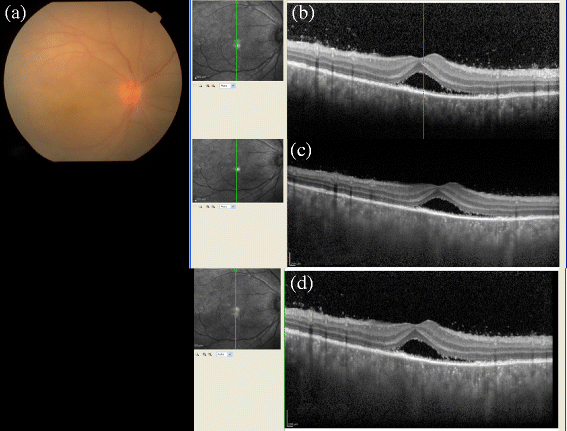

Supplement: Supplementary file 10 — Authors’ original file for figure 8 [file 12348_2014_18_MOESM10_ESM.gif]

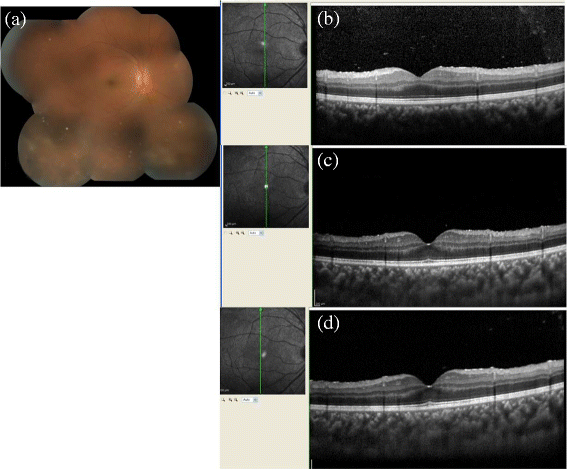

Supplement: Supplementary file 11 — Authors’ original file for figure 9 [file 12348_2014_18_MOESM11_ESM.gif]
